# Fasudil alleviates LPS-induced lung injury by restoring aquaporin 5 expression and inhibiting inflammation in lungs

**DOI:** 10.7555/JBR.31.20170024

**Published:** 2017-06-20

**Authors:** Jingjing Wang, Hui Kong, Jian Xu, Yanli Wang, Hong Wang, Weiping Xie

**Affiliations:** Department of Respiratory & Critical Care Medicine, the First Affiliated Hospital of Nanjing Medical University, Nanjing, Jiangsu 210029, China.; Department of Respiratory & Critical Care Medicine, the First Affiliated Hospital of Nanjing Medical University, Nanjing, Jiangsu 210029, China.; Department of Respiratory & Critical Care Medicine, the First Affiliated Hospital of Nanjing Medical University, Nanjing, Jiangsu 210029, China.; Department of Respiratory & Critical Care Medicine, the First Affiliated Hospital of Nanjing Medical University, Nanjing, Jiangsu 210029, China.; Department of Respiratory & Critical Care Medicine, the First Affiliated Hospital of Nanjing Medical University, Nanjing, Jiangsu 210029, China.; Department of Respiratory & Critical Care Medicine, the First Affiliated Hospital of Nanjing Medical University, Nanjing, Jiangsu 210029, China.

**Keywords:** acute lung injury, aquaporin 5, fasudil, fliud transport, NF-κB

## Abstract

Fasudil, a selective rho kinase (ROCK) inhibitor, has been reported to play a beneficial role in systemic?inflammation?in acute?lung injury, but its mechanism for ameliorating pulmonary edema and inflammation remains unclear. Using hematoxylin-and-eosin (H&E) staining, immunohistochemistry, enzyme-linked immunosorbent assay, quantitative real time PCR and Western blotting, we found that fasudil attenuated LPS-induced lung injury, decreased lung edema, and suppressed inflammatory responses including leukocyte infiltration and IL-6 production. Further, fasudil upregulated LPS-induced aquaporin 5 reduction and inhibited NF-κB activation in the lungs of mice. Our results suggest that fasudil could restore the expression of aquaporin 5 to eliminate LPS-induced lung edema and prevent LPS-induced pulmonary inflammation by blocking the inflammatory pathway. Collectively, blockade of the ROCK pathway by fasudil may be a potential strategy for the treatment of acute lung injury.

## Introduction

Acute lung injury (ALI) or acute respiratory distress syndrome (ARDS) is a leading cause of acute respiratory failure in critically ill patients. It is characterized by the disruption of the alveolar-capillary barrier which results in accumulation of protein-rich fluid in the alveolar cavity and leukocyte infiltration into the lung parenchyma. These pathological alterations lead to gas exchange abnormalities and hypoxemia^[1]^. Currently, there is no effective therapy for this critical illness, although a large number of patients are diagnosed with ARDS^[2]^. Despite improvement in supportive care, it has a high mortality among Intensive Care Unit patients^[1^,^3]^. Thus, it is an urgent need to search for promising therapeutic concepts that can reduce lung inflammation and permeability of the alveolar-capillary barrier in ALI.

Regulation of water movement in the lungs between the vascular, alveolar, and airway compartments is pivotal for normal lung function. Aquaporins (AQPs) are a family of integral membrane proteins and water-selective channels that mainly promotes osmotic water transport. To date, at least 4 types of AQPs (AQP1, AQP3, AQP4, and AQP5) are expressed in the airways and lungs^[4]^. Among these AQPs, AQP5 is the predominant water channel expressed in type Ι alveolar epithelial cells^[5^–^7]^. Previous investigators have shown that AQP5 deficiency in mice resulted in a 10-fold reduction of osmotic water permeability of the alveolar-capillary barrier in the lungs^[8]^ and the expression of AQP5 decreased in the model of radiation, viral infection and severe acute pancreatitis-related lung injury^[9^–^11]^. In addition, AQP5 deletion aggravates* Pseudomonas aeruginosa*-induced ALI^[12]^. Together, these studies suggest that AQP5 plays a pivotal role in maintaining normal water movement and inhibiting pulmonary inflammation in ALI.

ROCK signaling, which is involved in important biological functions including cell adhesion, migration and gene expression, can be activated by inflammatory stimuli such as TNF-α or/and LPS^[13]^. Several experimental researches suggest that fasudil, a ROCK selective inhibitor, is beneficial to ALI induced by lipopolysaccharide (LPS), ischemia or reperfusion-induced ALI^[14^–^15]^. However, the underlying mechanism was obscure. In this study, we explored the effects of fasudil on the expression of AQP5 and inflammation in the lungs of LPS-treated mice.

## Materials and methods

### Animal models of ALI

All experimental protocols were approved by the Institutional Animal Care and Use Committee of Nanjing Medical University and were in accordance with the guidelines of the National Institutes of Health. Male C57BL/6 mice (20~22 g, Changzhou Canvas Laboratory, Animal Corporation Ltd. China) were orally intubated with a sterile plastic catheter followed by anesthesia, and then challenged with LPS (5 mg/kg, *E. coli* 055:B5; Sigma) dissolved in 50 μL normal saline or saline as control. Fasudil (10 mg/kg, CHASE SUN, China) was given by intraperitoneal injection 1 h before LPS administration. Another group of mice without LPS instillation were also pretreated with fasudil to serve as controls for any side effects that might be attributed to fasudil. At 24 hours after LPS administration, mice were humanely killed by exsanguination under deep anesthesia to collect samples for analysis.

### Histological examination

To evaluate lung pathological changes of mice, the left upper lobes were fixed in 4% paraformaldehyde after dehydration in gradient ethanol and embedded in paraffin, sectioned at 3 μm, and stained with H&E. Images were taken under a light microscope (Leica, Germany) by two investigators blinded to group assignment. Ten random fields of each lung section were examined.

### Lung W/D ratio analysis

To evaluate water content in lungs, the wet weight of the right upper lobes was measured. Then, dry weight was obtained after the fresh lung lobes were desiccated in an oven at 70°C for 72 h. The lung water content was defined as the wet weight divided by the dry weight, namely lung W/D ratio.

### Immunohistochemistry

Lung sections were deparaffinized followed by heat-induced antigen retrieval with 0.01 mol/L citrate buffer and quenching of the endogenous peroxidase activity with 3% hydrogen peroxide. After 1-hour blockage with 3% BSA, the sections were incubated with primary antibodies of AQP5 (1:200, Santa Cruz Biotechnology) and CD11b (1:100, Cell Signaling Technology, Danvers, MA) overnight at 4°C and goat anti-rabbit secondary antibody (1:2,000, Proteintech) for 1 h at room temperature. The reactions were induced by a DAB substrate kit and hematoxylin as counterstain. Each slide was evaluated under a light microscope (Leica).

### ELISA

Concentrations of IL-6 in the bronchoalveolar lavage fluid (BALF), serum and lung tissues were measured with murine cytokine-specific ELISA kits (R&D Systems, Minneapolis, MN, USA) according to the manufacturer's instructions. Protein contents in the BALF were assayed by bicinchoninic acid (BCA) kit (Beyotime, China). IL-6 levels in the BALF and lung tissues were normalized by corresponding protein concentrations.

### Myeloperoxidase (MPO) analysis

MPO activities in the BALF and lung tissues were measured with a commercial test kit (Jiancheng Bioengineering Institute, Nanjing, China) following the manufacturer's directions. Protein concentrations were also measured with a BCA assay kit. MPO activities in the BALF and lungs were normalized by corresponding protein concentrations.

### Quantitative RT-PCR

Total mRNA of lung tissues was extracted with Trizol reagent (Gibco BRL, Grand Island, NY, USA). Reverse transcription was performed with 300 ng of total RNA with SYBR®Premix Ex TaqTM (TaKaRa, Japan). Quantitative RT-PCR was performed with Eppendorf Mastercylcer EP Realplex Real-time PCR System. Two-step RT-PCR was used to perform relative quantification of mRNA. The RT-PCR primer sequences are listed in *Table 1*. The 2^−ΔΔCt^ method was used to quantify mRNA expression relative to β-actin.

**Tab.1:** Sequences of primers used in this study

Gene	Forward primer	Reverse primer
*Rock2*	GAATTCATTCCTACCCTCTACCACTT	GGGCAGGTGGTGGCTTAA
*Aqp5*	GGCCACATCAATCCAGCCATTA	GGCTGGGTTCATGGAACAGCC
*Actb*	GGCATTGTTACCAACTGGGACGAC	CCAGAGGCATACAGGGACAGCACAG

### Protein extraction and Western blotting analysis

Frozen lung tissues were homogenized in a Protein Extraction Reagent (Thermo Scientific, Rockford), containing protease and phosphatase inhibitor cocktail. Twenty mg of protein was subjected to electrophoresis on 10% SDS-PAGE gels and transferred to PVDF membranes (Millipore). The membranes were blocked in TBST containing 5% skimmed milk for 1 h at room temperature and then incubated with primary antibodies against AQP5 (1:500, Santa Cruz Technology), occludin (1:1,000, Proteintech, Rosemont, IL), NF-κB p-p65/p65, p-iκκαβ/iκκαβ and ICAM-1 (1:1,000, Cell Signaling Technology) and goat anti-rabbit antibody (1:10,000, Proteintech), respectively. The immunoblots were developed with an ECL (Advansta). β-actin was used as loading control for each sample. Densitometric quantification was performed using Image Laboratory software to evaluate relative density of protein expressions against β-actin.

### Statistical analysis

For all statistical analysis, SPSS 18.0 software (SPSS Inc., Chicago, IL, USA) was used. All parametric data were compared by one-way ANOVA analysis followed by Turkey's-b test. Values were expressed as mean±SE. A value of *P*<0.05 was considered statistically significant.

## Results

### Fasudil attenuated LPS-induced lung injury

**Fig.1:**
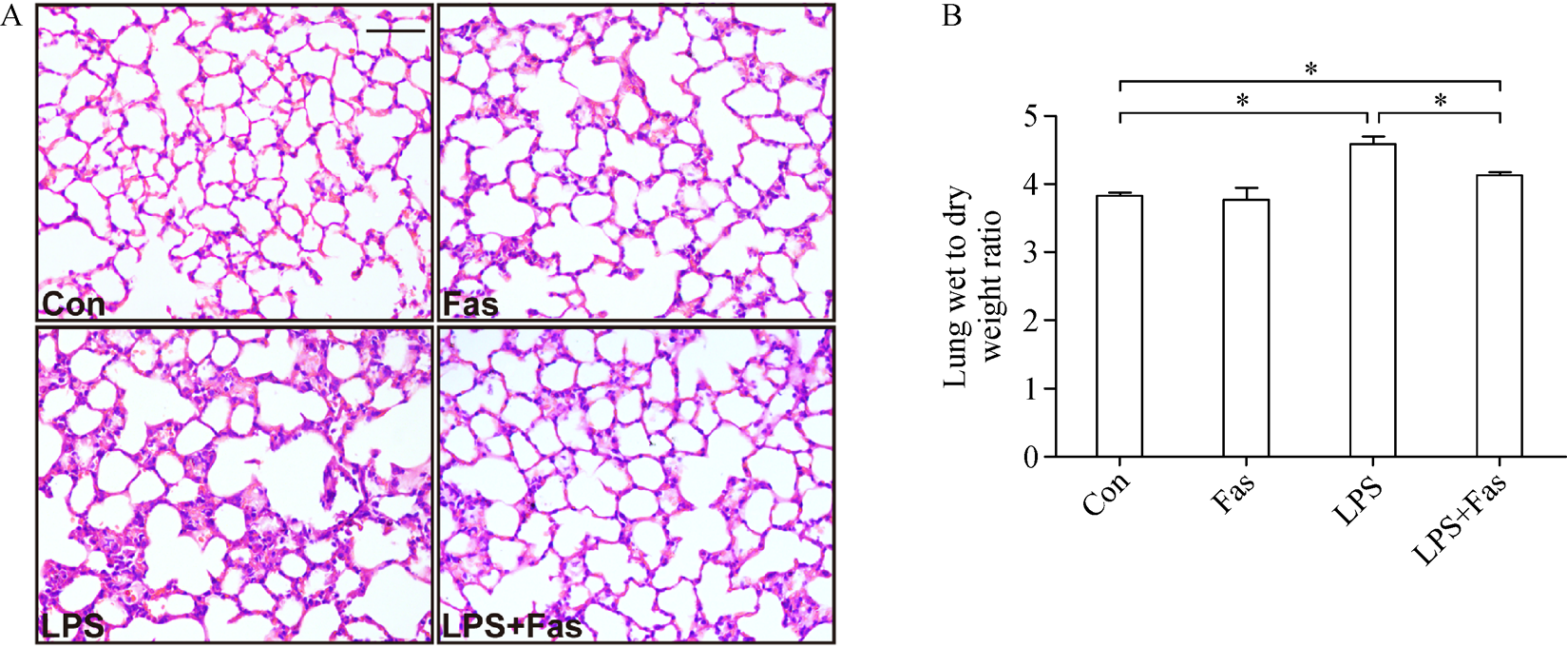
Fasudil attenuates LPS-induced pathological damage and lung W/D ratio in mice.

To characterize the effects of fasudil on LPS-induced lung injury, histological?analysis was performed by H&E staining. Lung sections from the saline group showed normal alveolar septa without pulmonary interstitial edema, while those from the LPS-treated mice had septal congestion in the lung tissues. However, fasudil significantly ameliorated lung injury induced by LPS (*Fig. 1A*). Accordingly, the W/D ratio demonstrated that fasudil predominantly reduced the high water content induced by LPS in the lungs of mice (*Fig. 1B*).

### Fasudil ameliorated lung inflammation caused by LPS

**Fig.2:**
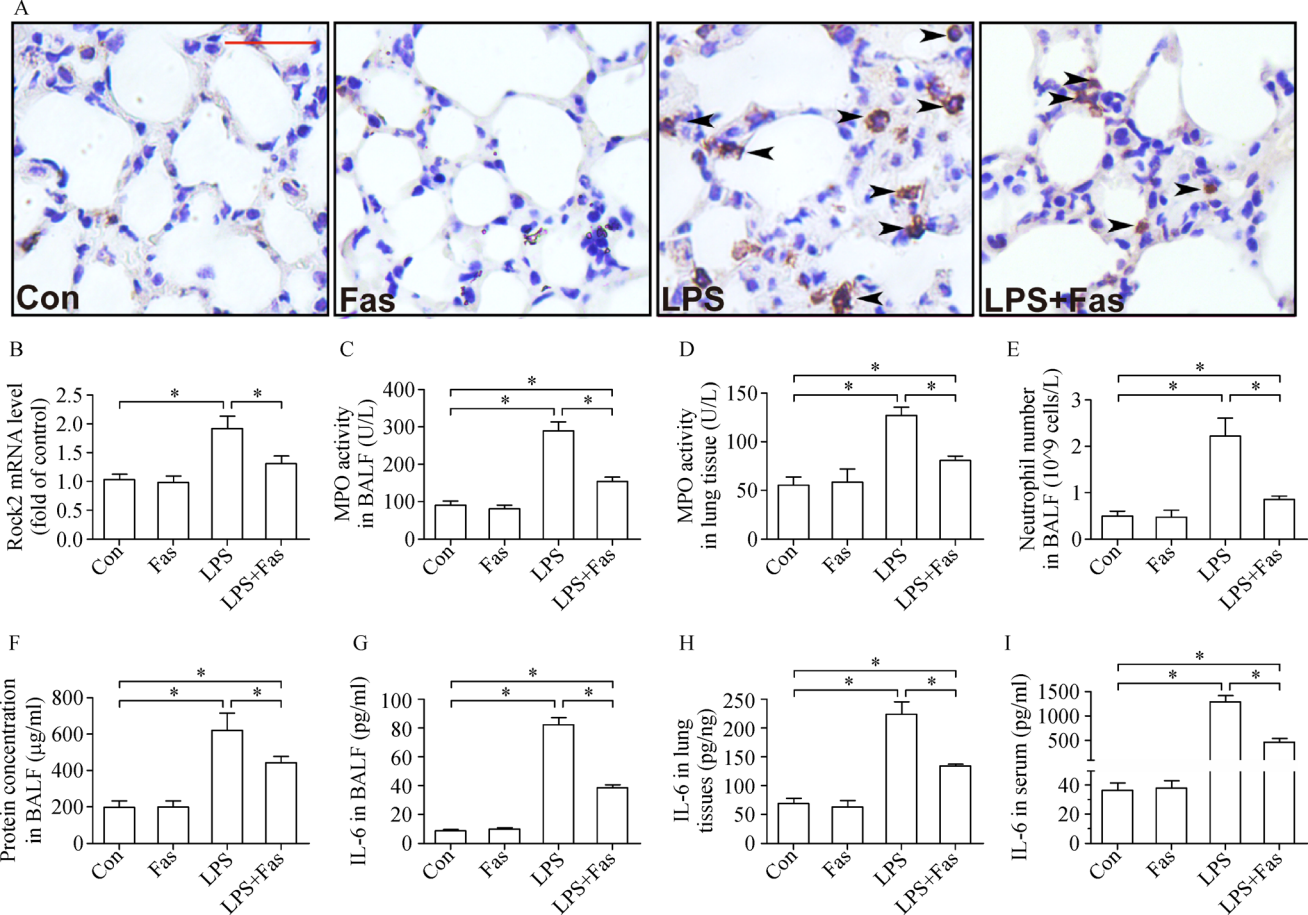
Fasudil mitigates LPS-induced lung inflammation.

Immunohistochemical analysis of CD11b, a marker of myeloid leukocytes, demonstrated that LPS induced a large number of leukocytes to infiltrate into the alveolar septa, which was significantly suppressed by fasudil (*Fig. 2A*). Further, fasudil blocked the mRNA transcription of Rock2 (*Fig. 2B*) and decreased MPO activities in the BALF and lung tissues (*Fig. 2C* and *D*) induced by LPS. Consistently, it inhibited the exudation of neutrophils and protein in the BALF (*Fig. 2E* and *F*) caused by LPS. Moreover, fasudil suppressed LPS-induced upregulation of IL-6 in the BALF, lung tissues as well as in peripheral blood (*Fig. 2G*–*I*).

### Fasudil reversed reduction of AQP5 and occludin induced by LPS

**Fig.3:**
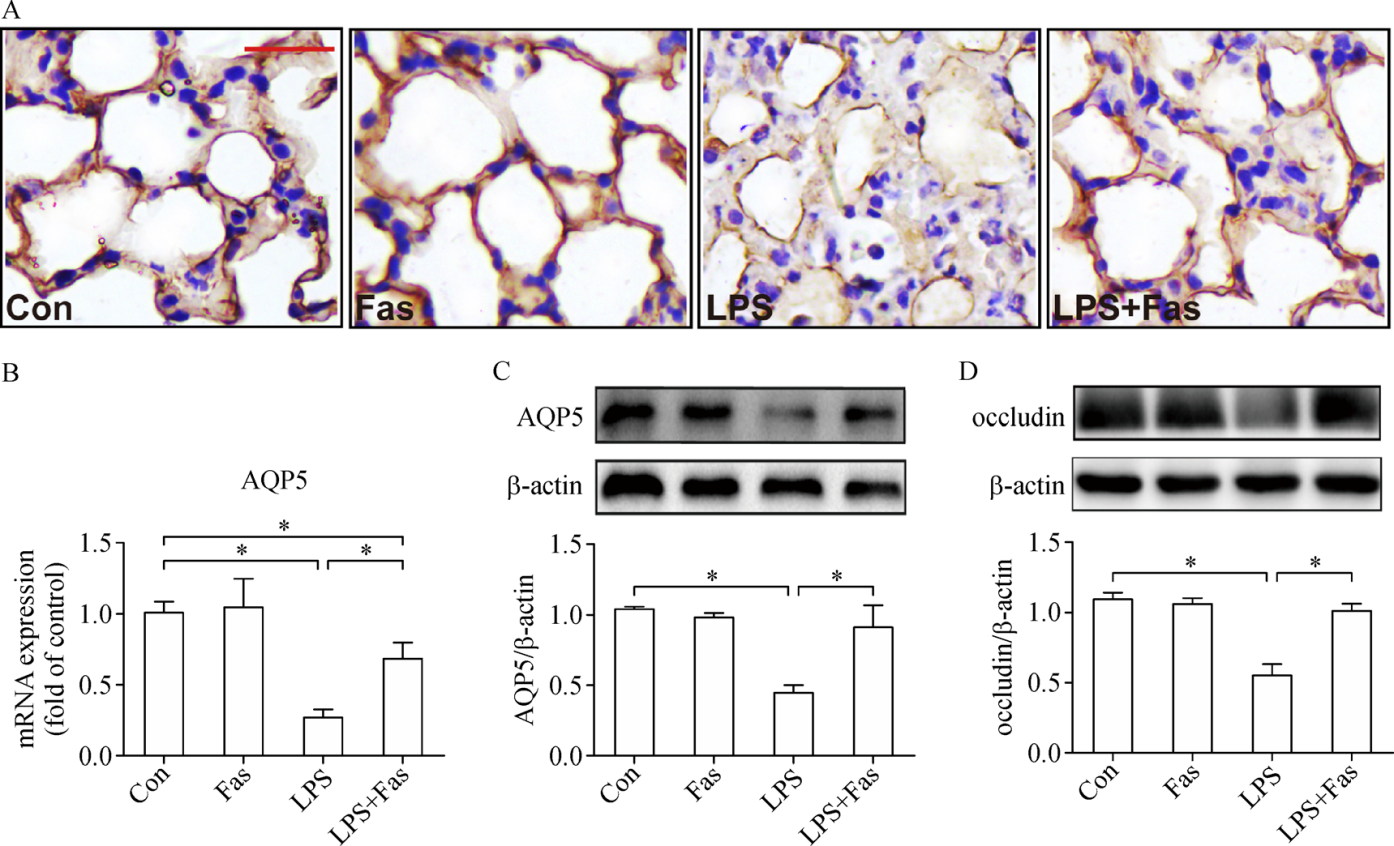
Fausil reverses LPS-induced reduction of AQP5 and occludin in the lungs of mice.

AQP5 is mainly distributed at the apical membrane of type Ι alveolar epithelial cells in the lungs. As shown in *Fig. 3A*, LPS challenge resulted in decrease of AQP5 in the alveolar epithelia of mice which was corrected by fasudil. To further validate this result, both quantitative and semiquantitative analysis of AQP5 expression by qRT-PCR and Western blotting were performed. Consistent with the results from immunohistochemistry, fasudil significantly increased or reversed LPS-induced downregulation of Aqp5 mRNA or protein in the lung tissues of mice (*Fig. 3B* and *C*).

Intercellular tight junction (TJ) occludin plays an important role in maintaining the integrity of the alveolar-capillary barrier and was examined in the study. As shown in *Fig. 3D*, LPS challenge led to a significant downregulation of occludin in the lungs, which, however, was restored by fasudil.

### Fasudil suppressed LPS-induced NF-κB activation and ICAM-1 overexpression

**Fig.4:**
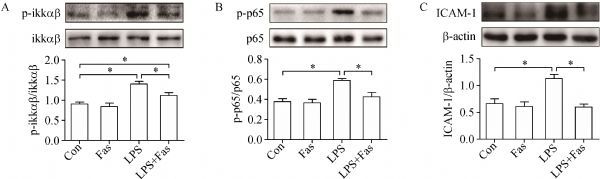
Fasudil inhibits the activation of NF-κB signal induced by LPS.

The NF-κB pathway is important in various inflammatory diseases. In this model of lung injury, LPS-induced NF-κB pathway activation as indicated by significant phosphorylation of iκκαβ (*Fig. 4A*) and NF-κB p-p65 (Ser535) (*Fig. 4B*) was partly or completely inhibited by fasudil. ICAM-1 is one of the most critical molecules regulated by NF-κB signaling and involved in leukocyte infiltration in ALI. Our results showed that LPS challenge induced significant overexpression of ICAM-1, but it was reversed by fasudil (*Fig. 4C*).

## Discussion

ALI or ARDS is characterized by hyperpermeability of the alveolar-capillary barrier and recruitment of inflammatory cells. In this study, we demonstrated that fasudil attenuated LPS-induced lung injury by inhibiting pulmonary edema formation and inflammation. Fasudil attenuated lung pathological damage, and suppressed the upregulation of ROCKα, neutrophil infiltration, protein concentration, MPO activities, IL-6 as well as NF-κB activation in the lungs of LPS-treated mice. In addition, it restored LPS-induced reduction of AQP5 and occludin in the lungs of mice.

Excessive fluid exudation and impaired fluid clearance in the alveolar spaces contribute to pulmonary edema in the development of lung injury. Previous studies suggest that dysfunction of alveolar fluid transport leads to poor prognosis in ARDS patients^[16]^. Conversely, augmenting the clearance of edema fluid and enhancing transepithelial sodium transport could be more effective for prevention or treatment of certain pulmonary edema^[17]^. Fluid transport in the lungs is mainly undertaken by the active pathway of Na, K-adenosine triphosphatase (Na, K-ATPase)^[18]^ and the passive pathway facilitated by AQPs^[5]^. AQP5 which is expressed at the apical membrane of type I alveolar epithelia and acinar epithelia in the submucosal glands osmotically drives water transport between alveolar airspaces and capillaries^[19]^. Downregulation of AQP5 in lung injury/edema is caused by various insults such as thoracic irradiation^[9]^, viral infection^[11]^ and endotoxin^[12]^, suggesting an important role of AQP5 under these conditions. Our data showed that LPS challenge induced the increase of W/D ratio and reduction of AQP5 in the lungs of mice. However, this effect of LPS on water content and the expression of AQP5 was offset by fasudil. Inhibiting ROCK by fasudil has been reported to attenuate endotoxin-induced lung injury, but the mechanism was not confirmed. Although there is a paucity of information to date regarding the relationship between ROCK and AQP5, our results indicate that fasudil ameliorates LPS-induced pulmonary edema through upregulating the expression of AQP5.

The integrity of epithelial barrier is also crucial in lung fluid homeostasis. TJs between the epithelia maintain the low permeability of normal epithelial cells to paracellular transport. It is a belt-like interconnected strand structure between alveolar epithelial cells and separates the apical and basolateral epithelium^[20]^. Two of the well-characterized proteins of TJs in the pulmonary epithelia are occludin, a transmembrane protein responsible for major barrier function and zonula occludens (ZO)-1, an intracellular protein linking occludin to cytoskeletal proteins^[21^–^22]^. Low concentrations of extracellular calcium and intracellular ATP and other biological factors including infection^[23]^, cytokines^[24]^ and reactive oxygen species (ROS)^[25]^ induce dissociation/disruption of TJs. In the present study, we showed that occludin was decreased by LPS instillation in the lungs of mice, but it was reversed by fasudil. ROCK inhibitor C3 transferase and Y-27632 partially reverse the reduction of occludin induced by human immunodeficiency virus Tat in brain microvascular endothelial cells^[26]^, indicating that ROCK is an upstream signaling of occludin. Combining these findings with our results, it is reasonable that fasudil attenuates lung edema by LPS *via* restoring the expression of occludin. Moreover, Kawedia *et.al* have demonstrated that AQP5 deletion leads to downregulation of TJ occludin, suggesting that AQP5 and occludin could coordinate to facilitate water transport^[27]^. Thus, it is suggested that fasudil ameliorates LPS-evoked pulmonary edema by increasing the expression of AQP5 and occludin.

It is widely accepted that lung inflammation is the major characteristic of lung injury. Apart from the classical proinflammatory cytokines IL-6, TNF-α, γ-IFN and TGF-β, inflammasome-regulated cytokines (such as caspase-1, IL-1β and IL-18)^[28]^, IL-17A^[29]^ and endocan^[30]^ are also important mediators of ALI or ARDS. All of the resident lung cells including the alveolar epithelia, vascular endothelia, alveolar macrophages, and fibroblasts as well as leukocytes participate in the inflammatory process^[31]^. Among them, the alveolar epithelia can release proinflammatory mediators, produce neutrophil chemotactic factors, and increase adhesion molecules induced by bacteria or endogenous factors IL-1β and TNF-α, while maintaining water balance^[32]^. Previous studies have shown that ROCK inhibition by fausdil prevents early lung inflammatory response induced by intratracheal instillation of LPS in mice through suppressing production of IL-6 and TNF-α^[33]^. Similarly, our data demonstrated that pretreatment with fasudil significantly inhibited leukocyte infiltration, MPO activities and cytokine production (IL-6) caused by LPS, indicating that fasudil could have a potent anti-inflammatory effect on LPS-induced lung injury.

NF-κB is a well-recognized signaling in LPS-induced lung inflammation which in turn promotes the transcription of cytokines, chemokines and adhesion molecules^[34]^. Previous studies have shown that LPS challenge-induced inflammation in the bronchial epithelia is impaired by inhibiting NF-κB activation with a constructed *I*κ*B*α mutant^[35]^. Moreover, ROCK results in significant NF-κB activation in inflamed intestinal mucosa^[36]^. Consistently, our results showed that ROCK blockade by fasudil inhibited LPS-induced phosphorylation of NF-κB p65 and iκκαβ. Thus, it could be suggested that ROCK signal participates in LPS-induced NF-κB activation.

It has been reported that* AQP5* knockout led to severe bacterial blood dissemination and lung injury in a mouse model of *Pseudomonas aeruginosa *infection^[12]^. Additionally, NF-κB inhibitors block the reduction of AQP5 in LPS-induced inflammation of salivary gland^[37]^. In our study, fasudil inhibited NF-κB activation accompanied by reduction of AQP5 induced by LPS. Combining the above findings with our results, fasudil could attenuate LPS-induced lung inflammation by inhibiting NF-κB activation and upregulating the expression of AQP5.

In conclusion, this is the first report about the effects of fasudil on the expression of AQP5 in LPS-induced ALI. Our study suggests that fasudil attenuated LPS-induced pulmonary edema *via* upregulating the expression of AQP5 and TJ protein occludin. Moreover, fasudil ameliorated LPS challenged-lung inflammation by inactivating NF-κB and increasing AQP5. However, the exact mechanism by which fasudil acts on the expression of AQP5 and the other effects of this protein in ALI needs to be further investigated.
